# Hydrogen bonding structure of confined water templated by a metal-organic framework with open metal sites

**DOI:** 10.1038/s41467-019-12751-z

**Published:** 2019-10-18

**Authors:** Adam J. Rieth, Kelly M. Hunter, Mircea Dincă, Francesco Paesani

**Affiliations:** 1 Department of Chemistry, Massachusetts Institute of Technology, 77 Mass. Ave., Cambridge, MA 02139 USA; 2 Department of Chemistry and Biochemistry, University of California San Diego, La Jolla, CA 92093 USA; 3 Materials Science and Engineering, University of California San Diego, La Jolla, CA 92093 USA; 4 San Diego Supercomputer Center, University of California San Diego, La Jolla, CA 92093 USA

**Keywords:** Materials chemistry, Metal-organic frameworks, Method development, Molecular dynamics, Statistical mechanics

## Abstract

Water in confinement exhibits properties significantly different from bulk water due to frustration in the hydrogen-bond network induced by interactions with the substrate. Here, we combine infrared spectroscopy and many-body molecular dynamics simulations to probe the structure and dynamics of confined water as a function of relative humidity within a metal-organic framework containing cylindrical pores lined with ordered cobalt open coordination sites. Building upon the agreement between experimental and theoretical spectra, we demonstrate that water at low relative humidity binds initially to open metal sites and subsequently forms disconnected one-dimensional chains of hydrogen-bonded water molecules bridging between cobalt atoms. With increasing relative humidity, these water chains nucleate pore filling, and water molecules occupy the entire pore interior before the relative humidity reaches 30%. Systematic analysis of rotational and translational dynamics indicates heterogeneity in this pore-confined water, with water molecules displaying variable mobility as a function of distance from the interface.

## Abstract (web summary)

The properties of water under confinement are significantly altered with respect to the bulk phase. Here the authors use infrared spectroscopy and many-body molecular dynamics simulations to show the structure and dynamics of confined water as a function of relative humidity within a metal-organic framework.

## Introduction

Due to the formation of frustrated hydrogen-bond (H-bond) networks, water confined within pores or at interfaces exhibits significantly altered physical properties compared to bulk water, with important implications for different fields, including chemistry^1,2^, biology^3–5^, and atmospheric science^6,7^. As a consequence of the unique structural and dynamical properties of frustrated H-bond networks, confinement of water gives rise to anomalous behavior, as inferred from measurements of various quantities, such as the dielectric constant^8^ and diffusion coefficient^9^. Significant progress has recently been made in developing a more complete picture of the water H-bonding structure^10^, especially due to the introduction of accurate many-body molecular models^11^. Nevertheless, a precise prediction of the properties of water across different phases and in different environments remains a challenge due to the dynamic nature of the H-bond network which results from the subtle balance between energetic, entropic, and nuclear quantum effects^11–13^.

As confinement increases, so too does the importance of interactions between the water molecules and the confining environment, with distinct consequences for the H-bonding structure^14,15^. Studies aiming to characterize the thermodynamic and dynamic properties of water in confinement have targeted various porous materials, such as hydrophobic carbon nanotubes^16–20^ and hydrophilic zeolites and silicas, as water containers^21^. It was found that water inside carbon nanotubes forms one-dimensional and tightly H-bonded chains, while ordered phases were identified in water confined in zeolites. On the other hand, water adsorbed on metal surfaces often displays well-defined patterns that are templated by the strength and anisotropy of the underlying water–metal interactions^22^. However, the uniformity of the hydrophobicity or hydrophilicity associated with these materials engenders mostly predictable water–substrate interactions that may differ significantly from those observed in heterogeneous environments, such as aquaporins and other structures found in biological systems^3^, where both hydrophilic and hydrophobic patches coexist and lead to a variety of competing H-bonding domains^1–7^.

**Fig. 1:**
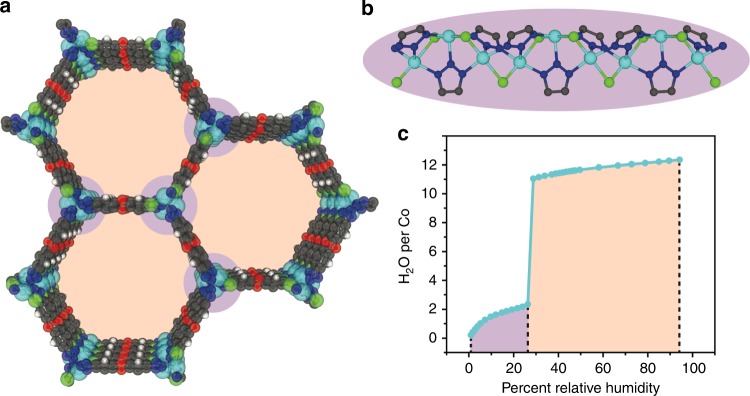
Structure and Water Adsorption of Co_2_Cl_2_BTDD. **a** Structure of Co_2_Cl_2_BTDD viewed down the *z*-axis. C – gray, H – white, O – red, N – dark blue, Cl – green, Co – light blue. Hydrophilic regions are indicated in purple and hydrophobic regions in orange. **b** Structure of the secondary building unit along the *z*-axis. **c** Water adsorption isotherm at 20 °C for Co_2_Cl_2_BTDD exhibiting complete pore hydration at 28% RH, data adapted from ref. ^30^

Metal-organic frameworks (MOFs) have recently received attention as water containers exhibiting tunable hydrophilicity of potential use in adsorption heat pumps^23–28^ and for atmospheric water harvesting^29,30^. In this regard, we posit that a MOF termed Co_2_Cl_2_BTDD (H_2_BTDD = bis(1*H*-1,2,3-triazolo[4,5-b],[4′,5′-i])dibenzo[1,4]dioxin)), which was recently investigated for its record reversible water uptake (Fig. 1), will provide a relevant crystalline analog for investigating the H-bonding structure of water in heterogeneous confinement^30^. Here we combine diffuse reflectance infrared Fourier transform spectroscopy (DRIFTS) measurements with many-body molecular dynamics (MB-MD) simulations^31–35^. Our analysis implicates the initial formation of distinct one-dimensional (1-D) chains of adsorbed water molecules bridging between the framework hydrophilic open metal sites as the critical step for pore filling in Co_2_Cl_2_BTDD. As the relative humidity (RH) increases, these 1-D chains template the subsequent formation of cylindrical water shells that extend along the hydrophobic pore channels and exhibit progressively faster rotational and translational mobility as a function of the distance from the pore surface.

## Results

### Infrared measurements of the water adsorption process

**Fig. 2:**
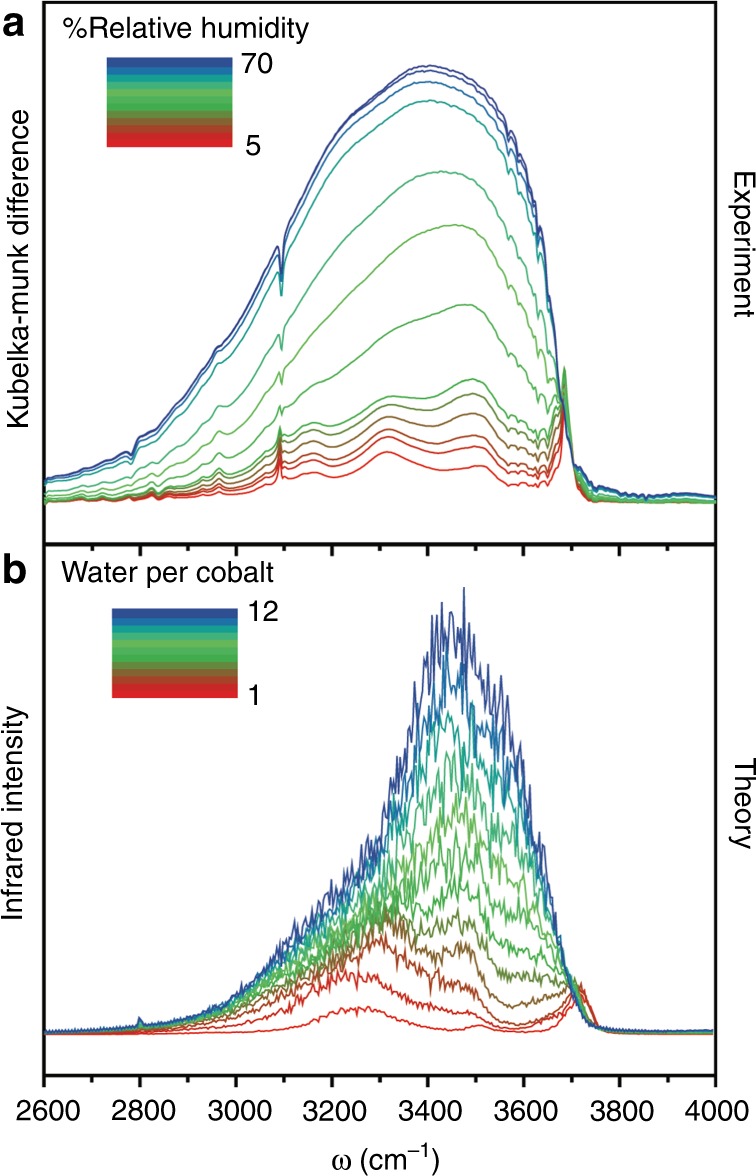
Experimental and Theoretical Infrared Spectra of Water in Co_2_Cl_2_BTDD. **a** Difference Diffuse-Reflectance IR spectra of the water OH-stretch region in Co_2_Cl_2_BTDD under variable RH conditions. **b** Calculated IR intensity using the MB-pol model of water in Co_2_Cl_2_BTDD ranging from one water molecule per cobalt (1) to twelve water molecules per cobalt (12)

Co_2_Cl_2_BTDD was synthesized as previously reported^36^, with a consistent powder X-ray diffraction pattern and N_2_ adsorption isotherm (Supplementary Figs. 6, 7). The structure exhibits hexagonal pores 2.2 nm in diameter linked by secondary building units consisting of infinite chains of cobalt chloride bridged by triazolate groups, wherein each nitrogen atom is ligated to a distinct Co^2+^ (Fig. 1a, b). DRIFTS spectra were measured at 20 °C under variable RH (Fig. 2b and Supplementary Fig. 8). With increasing RH, notable changes in the infrared spectrum appear around 600 cm^−1^ for the water librational mode, at 1600 cm^−1^ for the HOH bending mode, and near 3500 cm^−1^ for the water OH-stretching band (Supplementary Fig. 8)^37,38^. At low RH, the OH-stretching region displays several well-defined peaks, indicating that the water molecules experience distinct environments within the pore, which are non-equivalent on the IR timescale (Fig. 2a). The highest frequency peak at 3700 cm^−1^ can be attributed to the presence of free OH bonds, i.e., OH bonds that are not engaged in H-bonding^39^. Although free OH bonds are short-lived in bulk water^40^, they are present at the air/water interface^41,42^, which thus seems to provide a closer reference for water adsorbed at low RH in the Co_2_Cl_2_BTDD pores. The remaining series of peaks suggests a complicated interplay between water–framework and water–water interactions that lead to a broad range of H-bond strengths.

As the RH increases, the individual peaks coalesce into a single broad band resembling that of bulk water.

### Simulating water in confinement using the MB-pol model

A molecular-level interpretation of the origin and evolution of the different spectral features as a function of RH is obtained from classical MB-MD simulations carried out combining the MB-pol water model^31–35^, which has been shown to accurately predict the properties of water from the gas to the condensed phase^43^, with a flexible force field for Co_2_Cl_2_BTDD (see the Supplementary Methods, Supplementary Figs. 1–5, and Supplementary Tables 1–4 for details). All simulations were carried out at a temperature of 300 K (Supplementary Figs. 14, 15). To allow for direct comparison with the experimental data, all theoretical infrared (IR) spectra, which are calculated from the dipole-dipole time correlation function (see the Supplementary Methods for details), are red-shifted by 175 cm^−1^ in the OH-stretching region to account for zero-point energy effects that are neglected in classical MB-MD simulations, as discussed in detail in Refs. ^34,44^. Accounting for zero-point energy effects, good agreement is obtained with the experimental data (Fig. 2b and Supplementary Fig. 8), with the theoretical spectra reproducing the same distinct series of peaks at low RH and the emergence of a progressively broader band as the RH increases.

## Discussion

**Fig. 3:**
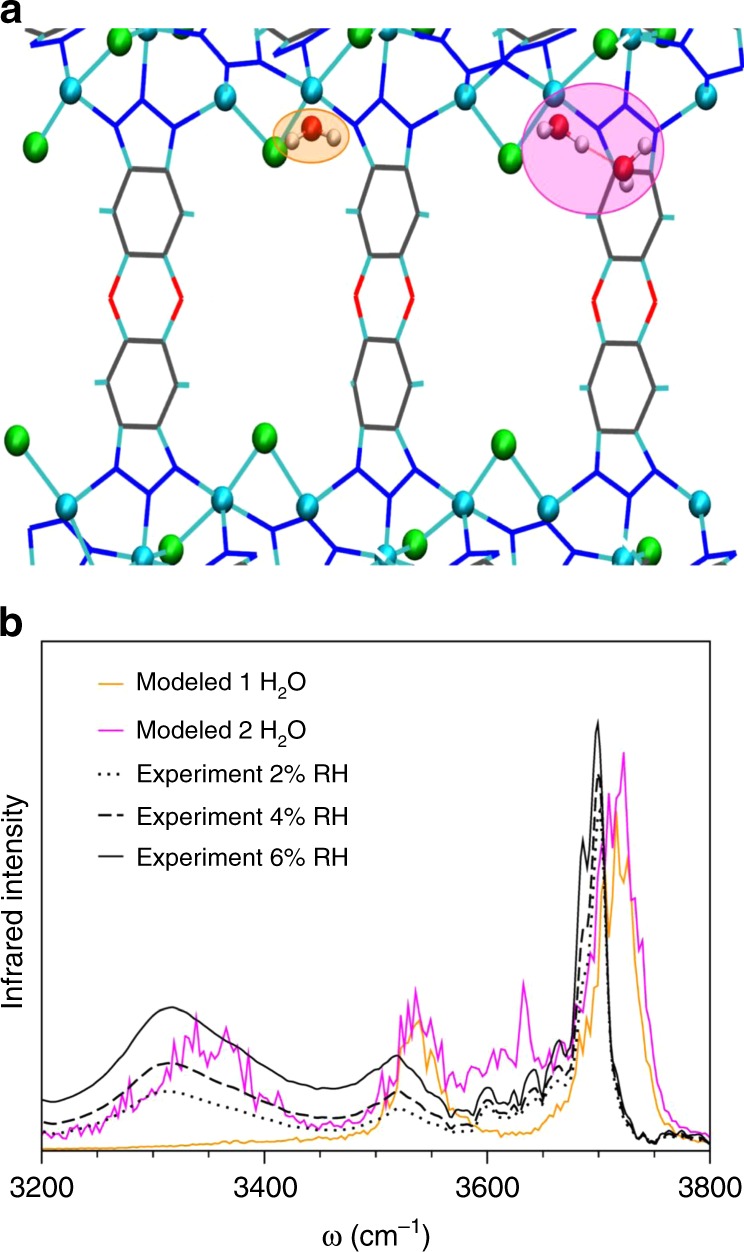
Water at Low Loadings in Co_2_Cl_2_BTDD. **a** Simulations with one (orange) and two (pink) water molecules. Distance shown along the *z-*axis is 24 Å. **b** Calculated IR OH-stretches of one (orange) and two (pink) water molecules with corresponding experimental DRIFTS spectra at RH 2% (dotted black), 4% (dashed black), and 6% (solid black)

To characterize the evolution of the H-bond network during pore filling, the results of separate MB-MD simulations carried out with only one and two water molecules in the simulation box are examined in Fig. 3a. The most favorable location of a single water molecule in the Co_2_Cl_2_BTDD pores corresponds to coordination with one of the open cobalt sites. In this configuration, the two OH bonds of the water molecule are not equivalent on a picosecond timescale due to different interactions with the framework. This results in two peaks at 3550 cm^−1^ and 3700 cm^−1^ (orange trace in Fig. 3b) that, while reminiscent of the symmetric and asymmetric stretches, correspond to two distinct OH-stretch vibrations (see Supplementary Fig. 9 for specific details, and Supplementary Tables 6–17 and Supplementary Figs. 14, 15 for details concerning simulation temperature at various water loadings). The peak at lower frequency is associated with the stretching vibration of the OH bond weakly interacting with a triazolate group of the framework while the peak at higher frequency is associated with the OH bond weakly interacting with the nearest chloride atom of the framework.

The addition of a second water molecule leads to the formation of a H-bonded dimer, with the first molecule remaining coordinated with the open cobalt site. MB-MD simulations indicate that the two OH bonds on the second water are relatively free to rotate (on a timescale of ~0.50 ps) due to lack of specific interactions with the framework. The decomposition of the theoretical IR spectrum in terms of individual OH-stretch contributions (pink trace in Fig. 3b) reveals that the second water molecule is primarily responsible for the peaks at 3550 cm^−1^ and 3700 cm^−1^. The cobalt-bound water is instead responsible for the emergence of the two new peaks at 3300 cm^−1^ and 3650 cm^−1^, with the first peak corresponding to the H-bonded OH-stretch and the second peak being associated with the non-H-bonded OH bond. Being relatively free to rotate, the non-H-bonded OH bond experiences a wide range of local environments and is thus found to also contribute to the peaks at 3550 cm^−1^ and 3700 cm^−1^ (see Supplementary Fig. 9 for specific details).

A nearly one-to-one correspondence is found in Fig. 3b between the theoretical IR spectra calculated for one and two water molecules and the experimental DRIFTS spectra measured at low RH. Based on the equilibrium water isotherm data (Fig. 1c), the experimental water uptake at 2, 4, and 6% RH corresponds to approximately 0.5, 0.9, and 1.2 water molecules per cobalt atom, respectively. Although water molecules interact more strongly with the open Co^2+^ sites, the presence of the spectral feature at 3300 cm^−1^, indicative of the water dimer at the cobalt site, thus implies that water begins forming localized H-bonded clusters seeded by cobalt-bound water molecules prior to full saturation of the open Co^2+^ sites.

**Fig. 4:**
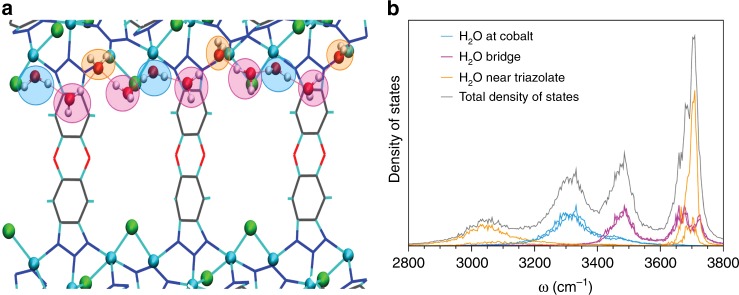
Chains of Water Bridge Between Cobalt Sites. **a** Structure of the one-dimensional water chain with water at cobalt (blue), water bridges (pink), and water near the triazolate (orange) highlighted. Distance shown along the *z-*axis is 24 Å. **b** Density of states calculated for individual hydrogen atoms

Simulating the adsorption of additional water molecules reveals the formation of 1-D water chains bridging between cobalt sites. These chains consist of three types of water molecules residing in three distinct local environments. Water molecules of the first type correspond to those bound to the open Co^2+^ sites (light blue in Fig. 4a), which engage in two H-bonds, one each to two adjacent water molecules of the second type. These second-type water molecules (pink in Fig. 4a) act as bridging molecules (similar to the second water molecule in Fig. 3a) by accepting and donating one H-bond and do not interact directly with the framework. Water molecules of the third type (orange in Fig. 4a) correspond to those that interact with the framework by pointing one OH bond to the π-system of the neighboring triazolate group, while accepting two H-bonds, one each from two adjacent bridging water molecules.

Further insights into the nature of the interactions between the framework and the water molecules residing in the three different local environments are gained by dissecting the density of states (DOS), corresponding to the theoretical spectra, into individual contributions associated with each type of water molecule along the 1-D chains. It is found that the cobalt-bound water molecules only contribute to the peak at 3300 cm^−1^ (light blue trace in Fig. 4b), which is a spectral feature characteristic of double donor H-bonded water molecules. Since these molecules are pinned to the open Co^2+^ sites and engage in two H-bonds, which are equivalent on the IR timescale, their mobility is highly frustrated (Supplementary Fig. 10). The water-bridging molecules contribute to the two peaks at 3500 and 3700 cm^−1^ (pink trace in Fig. 4b), which are associated with the stretching vibrations of the H-bonded and free OH bonds, respectively. Due to the absence of directional interactions with the framework, these molecules can easily switch H-bond partners and display significantly faster orientational mobility than the cobalt-bound molecules (Supplementary Fig. 10). Finally, the water-to-triazolate molecules contribute to the two bands at ~3050 and 3700 cm^−1^ (orange trace in Fig. 4b), with the lower frequency peak being associated with the stretching vibrations of the OH bonds pointing toward the π-systems of the triazolate groups and the higher frequency peak corresponding to the stretching vibrations of the other (free) OH bonds, respectively. Due to relatively stronger interactions with the triazolate groups, these water molecules exhibit rotational mobility that is intermediate between those displayed by cobalt-bound and bridging water molecules (Supplementary Fig. 10). The combination of the theoretical DOSs calculated for the three types of water molecules along the 1-D chains results in a vibrational lineshape (gray trace in Fig. 4b) that reproduces the main peaks of the experimental DRIFTS spectra measured below 30% RH, before the MOF pores become fully hydrated (Fig. 2a and Supplementary Table 5). Although connecting every open Co^2+^ site with water chains would require four water molecules per cobalt, the isotherm data indicate that pore hydration is initiated at 2.3 water molecules per cobalt (Fig. 1c). This suggests that disconnected 1-D chains form in various locations within the framework before all cobalt sites are saturated, which is supported by our MB-MD simulations showing that 1-D chains bridging multiple cobalt sites begin to appear at a loading of two water molecules per cobalt.

**Fig. 5:**
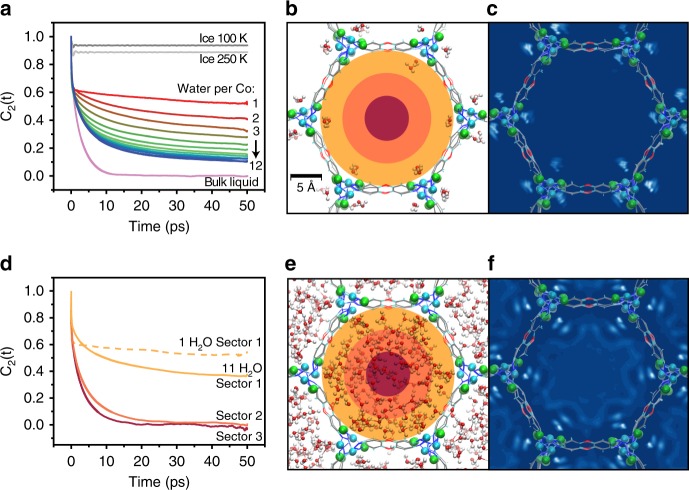
Dynamics of Water in Co_2_Cl_2_BTDD. **a** Orientational correlation functions calculated from MB-MD simulations carried out for various loadings of water inside the Co_2_Cl_2_BTDD pores. Also shown for reference are the corresponding orientational correlation functions calculated from MB-MD simulations of liquid water (pink) and ice (gray). **b** Snapshot from an MB-MD simulation of water in Co_2_Cl_2_BTDD for a loading of 1 H_2_O/Co^2+^. The three concentric colored sectors are defined according to their distance from the pore surface: Sector 1 from 0–4 Å (dark yellow), Sector 2 from 4–8 Å (orange), and Sector 3 from 8–12 Å (red). **c** Two-dimensional (2-D) density map of water calculated from MB-MD simulations carried out for a loading of 1 H_2_O/Co^2+^ (lighter colors correspond to regions with higher water density). **d** Orientational correlation functions calculated for water in the three different sectors of the pore at either 1 H_2_O/Co^2+^ (dashed line) or 11 H_2_O/Co^2+^ (solid lines). **e** Snapshot of an MB-MD simulation of water in Co_2_Cl_2_BTDD for a loading of 11 H_2_O/Co^2+^ (the three concentric colored sectors are defined as in **b**. **f** 2-D density map for a loading of 11 H_2_O/Co^2+^ (lighter colors correspond to regions with higher water density)

At higher water loadings, the 1-D water chains bridging the hydrophilic open Co^2+^ sites act as nucleators for the pore filling process, templating the formation of concentric cylindrical shells that extend along the hydrophobic pore channels. As the water loading increases, the MB-MD simulations indicate that the water molecules become, on average, more mobile (Fig. 5a). Because the orientational correlation functions reflect the extent of molecular rotation over time, this suggests the emergence of liquid-like behavior. However, at the experimental maximum loading of 12 H_2_O/Co^2+^, the average orientational mobility of the water molecules in the Co_2_Cl_2_BTDD pores remains intermediate between that calculated for ice and bulk water. A similar slowdown was predicted for water adsorbed in MIL-53^45^.

The evolution of the dynamical behavior of water adsorbed in Co_2_Cl_2_BTDD as a function of RH can be further characterized by analyzing the variation of the water mobility from the surface to the center of the pore. To this purpose, the water molecules along the MB-MD trajectories are classified based on their distances from the surface of the pore and thus assigned to three concentric cylindrical sectors (shown in dark yellow, orange, and red in Fig. 5b, e), with a width of 4.0 Å each.

As discussed above, at low RH corresponding to a loading of 1 H_2_O/Co^2+^, the water molecules are primarily coordinated to the open cobalt sites, although there is not a one-to-one correspondence, and Co^2+^-pinned water dimers and trimers also form within the pores as inferred by the DRIFTS lineshapes. These water molecules thus reside in sector 1, providing an outer shell of H-bonding sites that effectively template the development of the H-bonding structure inside the pores at high RH (Fig. 5b, c). Water molecules enter sector 2 at a loading of 2 H_2_O/Co^2+^ and start filling sector 3 at a loading of 7 H_2_O/Co^2+^ (Fig. 5d and Supplementary Figs. 11, 12). At the loading of 11 H_2_O/Co^2+^, the water molecules fill the pore completely although they tend to cluster around the open cobalt sites as shown in Fig. 5e, f. Similar templating effects of the framework on adsorbed water were predicted from computer simulations of water in [Zn(L)(X)] (L = 3-methyl-2-(pyridin-4- ylmethylamino)-butanoic acid and X = Cl and Br)^46^, and MOF-74^47^. Analysis of both orientational and translational mobility shows that water molecules occupying different sectors exhibit significantly different dynamical behavior (Table 1 and Supplementary Figs. 11, 12). While water molecules in sector 1 reorient very slowly and are effectively translationally immobile, being directly coordinated with the Co^2+^ atoms or H-bonded to the Co-bound molecules, water molecules in sectors 2 and 3 display progressively faster dynamics. In particular, water molecules at the center of the pore (sector 3) reorient on a timescale of ~4.4 ps and diffuse along the pore (*z* direction) by 0.24 Å^2^ ps^−1^, which suggests dynamical behavior similar to liquid water (Table 1). The overall difference in the orientational relaxation times and diffusion coefficients of Table 1 displays the dynamical heterogeneity that exists between the different sectors due to their distance from the pore surface. Additionally, it should be noted that, due to confinement, water mobility along the *x* and *y* directions is systematically lower than along the *z* direction.

**Table 1:** Dynamics of water in Co_2_Cl_2_BTDD orientational relaxation time (*τ*_2_), calculated from ${\rm{C}}_2(t)={\rm{Ae}}^{-t/{\rm{T}}_2}$, along with the total diffusion coefficient (D_tot_), diffusion coefficient along the *xy*-plane (D_*xy*_), and diffusion coefficient along the *z* direction (D_*z*_) for 11 H_2_O/Co^2+^ with standard deviations. Experimental data for *τ*_2_ and D_tot_ in bulk water are taken from refs. ^49,50^, respectively. See the Supplementary Methods for specific details about the calculation of orientational relaxation time and diffusion coefficients

	*τ*_2_ (ps)	D_tot_ (Å^2^ · ps^−1^)	D_*xy*_ (Å^2^ · ps^−1^)	D_*z*_ (Å^2^ · ps^−1^)
Sector 1	>70	0.03 ± 0.01	0.02 ± 0.01	0.04 ± 0.01
Sector 2	8.3 ± 1.8	0.11 ± 0.03	0.09 ± 0.03	0.15 ± 0.04
Sector 3	4.4 ± 0.9	0.19 ± 0.05	0.16 ± 0.05	0.24 ± 0.08
Exp. Bulk water	2.5	0.23		

No standard deviations were calculated for the orientational relaxation time in Sector 1 since it was only possible to determine that the relaxation time is longer than 70 ps

In addition to the dynamical heterogeneity exhibited by water molecules in different pore environments, structural parameters reveal increasingly frustrated H-bond networks closer to the pore surface. At the maximum loading of 12 H_2_O/Co^2+^, Supplementary Fig. 13 shows that the distribution of the tetrahedral order parameter, q_tet_, a metric of the local structure of the water H-bond network, displays two distinct features—a dominant one with a maximum at q_tet_ ≈0.4, indicating less tetrahedrality and suggesting environments with interfacial character, and a second feature with a maximum at q_tet_ ≈ 0.8, representative of liquid-like environments^48^. The structural and dynamical heterogeneity exhibited by water at the maximum loading thus mirrors the water adsorption process observed by DRIFTS, as the sites with the highest water density, the least tetrahedral H-bonding network, and the slowest mobility in the full pore correspond with the sites of initial water adsorption near the metal sites.

Water confined in Co_2_Cl_2_BTDD pores exhibits similarities and differences with water adsorbed on surfaces. For example, water at metal surfaces tends to display long-range order^51^. Although at low RH, water inside Co_2_Cl_2_BTDD displays a similar order due to coordination to the open cobalt sites, higher RH disrupts the long-range order, and water molecules display progressively liquid-like behavior as they approach the center of the MOF pores. This is reflected in the broadening of the OH-stretch vibrational lineshapes towards lower frequencies characteristic of H-bond networks.

In summary, water adsorbed in Co_2_Cl_2_BTDD displays heterogeneous structural and dynamical behavior which varies as a function of both RH and distance from the pore surface. By directly connecting adsorption isotherms with the evolution of IR spectra of water inside MOF pores as a function of RH, the foregoing combined experimental and theoretical approach provides detailed insights into the molecular mechanisms that determine water adsorption in porous materials exhibiting both hydrophilic and hydrophobic regions. These mechanistic insights can contribute to the design of next-generation porous materials for water harvesting. Fundamentally, our approach advances the understanding of water structure and dynamics within amphipathic confined and interfacial environments which are widespread in biology, atmospheric science, and chemistry.

## Methods

### Synthesis

Co_2_Cl_2_BTDD was synthesized and activated according to a previously published procedure^36^. Briefly, 200 mg H_2_BTDD^3^ (0.75 mmol) was dissolved in 200 mL N,N’-dimethylformamide (DMF) with heating, then cooled to room temperature. Separately, 1.5 mmol (2 eq.) cobalt chloride hydrate was dissolved in 200 mL ethanol and 4 mL concentrated hydrochloric acid. The clear solutions were combined, capped, and heated to 65 °C in an oven for 10 days. The resulting solids were filtered, washed with DMF and methanol. Solvent exchange of DMF was carried out by Soxhlet extraction with methanol for approximately 48 h. The materials were then activated under dynamic vacuum at 150 °C for 24 h.

### Spectroscopic measurements

Diffuse reflectance infrared Fourier transform spectroscopy (DRIFTS) measurements were performed using a Bruker Tensor 37 IR spectrometer equipped with a liquid nitrogen cooled mercury cadmium telluride detector and a Pike DiffusIR accessory. A sample of Co_2_Cl_2_BTDD, pre-activated at 150 °C under vacuum to remove all solvents, was diluted with KBr in a ratio of approximately 1:5 (MOF:KBr) in an argon-filled glovebox. The resulting solid solution was then packed into a ceramic cup and sealed in the DiffusIR cell. The cell was brought out of the box, and a static dry spectrum was recorded with the cell sealed. Two gas streams of flowing argon (UHP grade 5.0, Airgas), one wet (bubbled through a fine frit through MilliQ H_2_O) and one dry, were each flow controlled using mass flow controllers (MFCs), and joined together at a T fitting before connecting to the DRIFTS cell. The wet stream and dry stream were controlled via the MFCs to change relative humidity (RH) every 20 min (a time period previously demonstrated to result in saturation of the IR spectrum at all loadings). The MFCs were controlled such that the total flow rate was constant at 1 liter per minute (LPM) (e.g. for 40% RH, 0.4 LPM wet, 0.6 LPM dry). The temperature for all measurements was 20 °C. Spectra were recorded at the end of the period at which the sample atmosphere was at each RH, every 20 min. Data was transformed using the Kubelka-Munk function^52^. The static dry spectrum was subtracted from the humid measurements in all cases.

### Molecular dynamics simulations

All simulations utilized the many-body potential energy function (MB-pol) to describe water, which is built upon a many-body expansion of the interaction energy for water^31–33^. MB-pol has been previously shown to accurately reproduce the properties of water from the gas to the condensed phase^35^. The framework atoms of Co_2_Cl_2_BTDD were modeled with a flexible force field consisting of point charges (details in the Supplementary Methods). In simulating Co_2_Cl_2_BTDD, the first configuration utilizes one water molecule per cobalt atom (1, corresponding to 54 water molecules), and in each subsequent simulation at each loading, an additional water molecule is added per cobalt atom (2, 3, etc.). The initial configurations for each loading were generated using Packmol^53^, adding the specific number of water molecules to 756 MOF atoms. Classical many-body molecular dynamics (MB-MD) simulations were performed using in-house software based on the DL_POLY_2 simulation package^54^, which was modified to include the MB-pol potential energy function^31–33^. All simulations were carried out for a system consisting of 1 × 1 × 3 primitive cells under periodic boundary conditions with cell dimensions 38.6590 Å, 33.4793 Å, and 25.6914 Å and angles 90°, 90°, and 120° along the *x*, *y*, and *z* dimensions, respectively. Each system was equilibrated through a constant volume and constant temperature (NVT) canonical ensemble at 300 K for 10 ps, and dynamical information was obtained through a constant volume and constant energy (NVE) microcanonical ensemble for 50 ps where the temperature remained stable around 300 K. Constant pressure and constant temperature (NPT) simulations at 1.0 atm and 300 K were also performed to investigate the flexibility of the framework throughout the simulation. During the NPT simulations, all three cell dimensions vary by ~4.5% with average values of 36.6955 Å, 31.7789 Å, and 24.3865 Å along the *x, y*, and *z* dimensions, respectively. While the cell size varies slightly, the framework remains constant in size throughout the simulations. Twenty independent MB-MD trajectories starting from different initial configurations were performed for each loading with a time step of 0.2 fs. The equations of motion were propagated according to the velocity-Verlet algorithm, and the temperature was maintained at 300 K by a Nosé-Hoover chain of four thermostats^55^. Short-range interactions were truncated at an atom–atom distance of 9.0 Å, and the electrostatics were calculated using the Ewald sum^56^. Standard long-range electrostatic interactions as implemented in DL_POLY_2 were applied to Lennard-Jones potentials to account for errors due to the truncation at 9 Å^54^. Cross interactions between water and the MOF were derived from Lorentz-Berthelot mixing rules.

## Supplementary information

Supplementary Information

## Data Availability

Any data generated and analyzed for this study that are not included in this Article and its Supplementary Information are available from the authors upon request.

## Code availability

The MB-pol water model used in this study is available in OpenMM (http://paesanigroup.ucsd.edu/software/mbpol_openmm.html) and i-PI (http://paesanigroup.ucsd.edu/software/mbx.html). All computer codes used in the analysis presented in this study are available from the authors upon request.
