# Supplementary material for: Hydrogen bonding structure of confined water templated by a metal-organic framework with open metal sites
Source: Nat Commun. 2019 Oct 18;10:4771. doi: 10.1038/s41467-019-12751-z (PMC6802106; doi:10.1038/s41467-019-12751-z)
Supplement: Supplementary file 1 — Supplementary Information [file 41467_2019_12751_MOESM1_ESM.pdf]

Supplementary Information

Hydrogen Bonding Structure of Confined Water Templated by a Metal-Organic Framework with Open Metal Sites

Rieth et al.

Supplementary Methods

$CoCl_2$•$6H_2O$ (99.9%, Alfa Aesar), HCl (32-35%, BDH – VWR Analytic), methanol (99.9%, VWR), and *N*,*N*-dimethylformamide (99.8%, Millipore) were used as received.

Powder X-ray diffraction (PXRD) patterns were recorded with a Bruker Advance II diffractometer equipped with a θ/2θ Bragg-Brentano geometry and Ni-filtered CuKα radiation ($K\alpha_1$ = 1.5406 Å, $K\alpha_2$ = 1.5444 Å, $K\alpha_1$/ $K\alpha_2$ = 0.5). The tube voltage and current were 40 kV and 40 mA, respectively. Samples for PXRD were prepared by placing a thin layer of the appropriate material on a zero-background silicon crystal plate.

Nitrogen adsorption isotherms were measured by a volumetric method using a Micromeritics ASAP 2020 gas sorption analyzer. A typical sample of ca. 40 mg of metal-organic framework, pre-activated at 100°C to remove all residual solvent, was transferred in an Ar filled glovebox to a pre-weighed analysis tube. The tube with sample inside was weighed again to determine the mass of the sample. The tube was capped with a Micromeritics TranSeal, brought out of the glovebox, and transferred to the analysis port of the gas sorption analyzer. Free space correction measurements were performed using ultra-high purity He gas (UHP grade 5, 99.999% pure). Nitrogen isotherms were measured using UHP grade nitrogen. All nitrogen analyses were performed using a liquid nitrogen bath at 77 K. Oil-free vacuum pumps were used to prevent contamination of sample or feed gases.

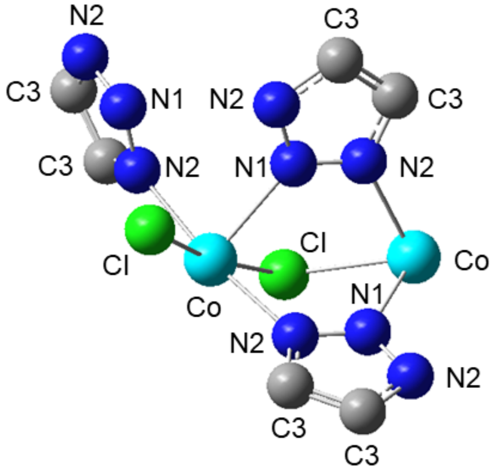

Supplementary Figure 1. Force field development for Co_2Cl_2BTDD Atom labels used in the definition of the force field parameters for Co_2Cl_2BTDD.

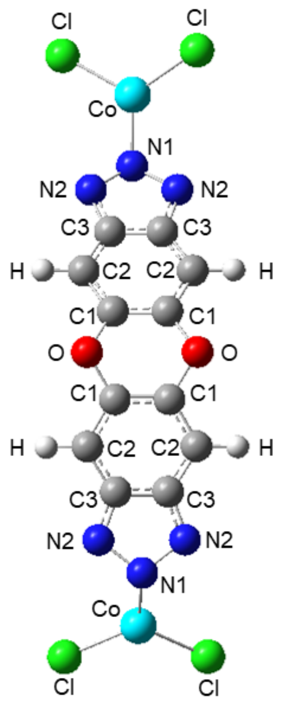

Supplementary Figure 2. Force field development for Co_2Cl_2BTDD Atom labels used in the definition of the force field parameters for Co_2Cl_2BTDD.

The General Amber Force Field (GAFF) was used to model the intramolecular interactions of the ligand,[8] while the force field parameters associated with the description of all bonds, angles, and dihedrals containing the Co centers were obtained from potential energy scans along the reduced molecular model. Similarly, potential energy scans of a single water molecule with a cobalt atom, consisting of 91 distorted configurations obtained by displacing the water molecule by 0.1 Å along the *z* dimension away from the Co atom, were performed. These calculations were carried out with Gaussian 09[9] at the DFT level using the ωB97X-D functional[10] in combination with the LanL2DZ basis set.[11-14] The actual fits for the ligand were performed on the reduced molecular model with a genetic algorithm[15] on 33 distorted configurations obtained by displacing the Co atom by 0.04 Å, 0.05 Å, and 0.05 Å along the *x, y,* and *z* dimensions, respectively. To derive the cross interactions between the ligand and water, the Lorentz-Berthelot mixing rules were used with the TIP4P/2005 water model,[16] which is the closest point-charge model to MB-pol.

The complete list of parameters of the force field for Co_2Cl_2BTDD is reported in Supplementary Tables 1-4.

Supplementary Table 1. Force field parameters for Co_2Cl_2BTDD. Electrostatic and Lennard-Jones potentials.

Atom name	Atom type	Charge	ε (kcal · mol^{-1})	σ / 2 (Å)
Co	Co	1.150297	0.0015	1.0842
Cl	Cl	-0.107034	0.0128	2.4152
C1	C	0.302577	0.0860	1.6998
C2	C	-0.577578	0.0860	1.6988
C3	C	0.230110	0.0860	1.6988
H	H	0.325060	0.0150	1.2998
O	O	-0.371140	0.1700	1.5000
N1	N1	-0.359101	0.1700	1.6250
N2	N2	-0.436680	0.1700	1.6250

Supplementary Table 2. Force field parameters for Co_2Cl_2BTDD. Bond potentials.

Bond type (Harmonic)	K_{ij} (kcal mol^{-1} Å^2)	r_0 (Å)
Co-Cl	109.5	2.383
Co-N1	20.2	1.903
Co-N2	197.8	1.871
N1-N2	973.6	1.379
N2-C3	985.8	1.336
C1-C2	956.8	1.387
C2-C3	956.8	1.387
C1-C1	956.8	1.387
C3-C3	956.8	1.387
C2-H	688.6	1.087
C1-O	744.8	1.373

Supplementary Table 3. Force field parameters for Co_2Cl_2BTDD. Bending potentials.

Angle type (Harmonic)	K_{ijk} (kcal mol^{-1} rad^2)	θ (°)
N2-Co-N1	163.564	93.095
Cl-Co-N1	153.249	98.125
N2-Co-N2	119.119	175.556
Cl-Co-N2	28.043	92.404
Cl-Co-Cl	119.765	167.206
N2-N1-Co	89.310	119.927
N2-N1-N2	52.062	110.750
C3-N2-Co	239.584	121.307
N1-N2-Co	84.987	123.028
Co-Cl-Co	635.579	80.771
C1-O-C1	126.620	119.950
C1-C1-O	139.580	119.200
C2-C1-O	139.580	119.200
C1-C2-H	96.920	120.010
C3-C2-H	96.920	120.010
C3-C3-C2	134.360	119.970
C3-C2-C1	134.360	119.970
C2-C1-C1	134.360	119.970
C3-C3-N2	140.280	119.720
C2-C3-N2	140.280	119.720
C3-N2-N1	151.886	112.100

Supplementary Table 4. Force field parameters for Co_2Cl_2BTDD. Torsion potentials.

Dihedral type	K_{ijk} (kcal mol^{-1})	χ (°)	N
N1-Co-N2-N1	0.0000	180.000	1
Cl-Co-N1-N2	0.0000	180.000	1
N2-Co-N1-N2	0.0000	180.000	1
C1-C1-O-C1	0.4500	180.000	2
C2-C1-O-C1	1.8125	180.000	2
C1-C1-C2-C3	1.8125	180.000	2
C1-C2-C3-C3	1.8125	180.000	2
C2-C3-C3-C2	1.8125	180.000	2
N2-C3-C3-N2	1.8125	180.000	2
O-C1-C1-O	1.8125	180.000	2
O-C1-C2-C3	1.8125	180.000	2
O-C1-C1-C2	1.8125	180.000	2
C3-N2-N1-N2	2.0000	180.000	2
C3-C3-N2-N1	2.4000	180.000	2
O-C1-C2-H	1.8125	180.000	2

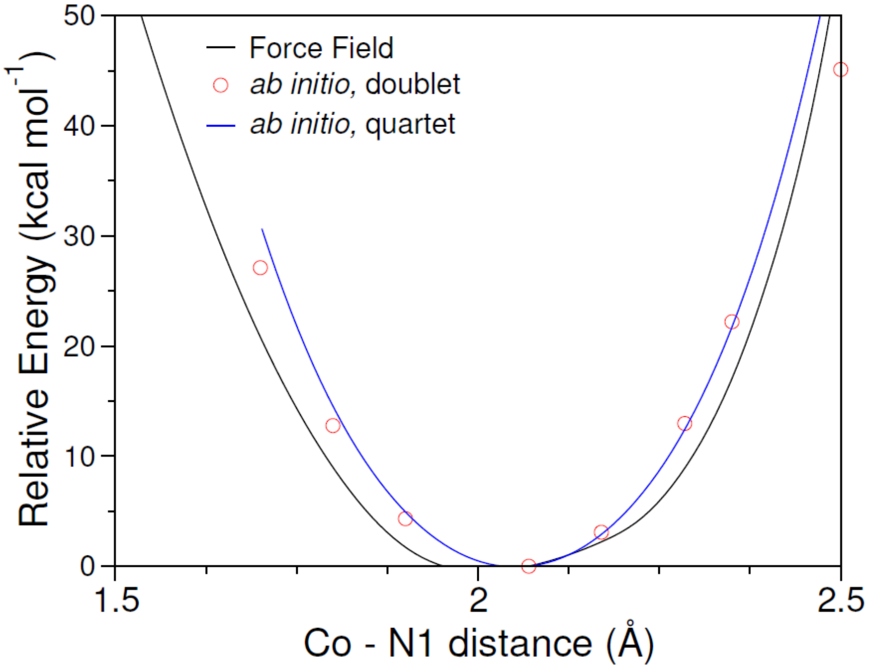

Supplementary Figure 3. Force field development for Co_2Cl_2BTDD Comparison of force field and *ab initio* DFT energies using the *ω*B97X-D functional in combination with the LanL2DZ basis set of both the doublet and quartet spin states on the Co^{2+} atom of the reduced molecular model as in **Supplementary Figure 1**. The Co^{2+} was scanned relative to the N1 atom of the framework for relative energies up to 50 kcal mol^{-1}.

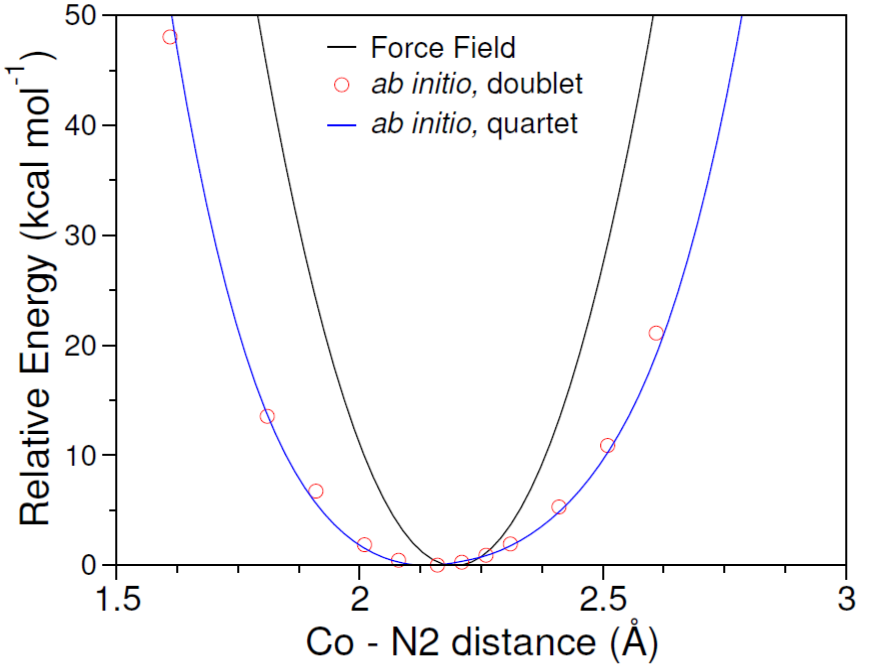

Supplementary Figure 4. Force field development for Co_2Cl_2BTDD Comparison of force field and *ab initio* DFT energies using the *ω*B97X-D functional in combination with the LanL2DZ basis set of both the doublet and quartet spin states on the Co^{2+} atom of the reduced molecular model as in **Supplementary Figure 1**. The Co^{2+} was scanned relative to the N2 atom of the framework for relative energies up to 50 kcal mol^{-1}.

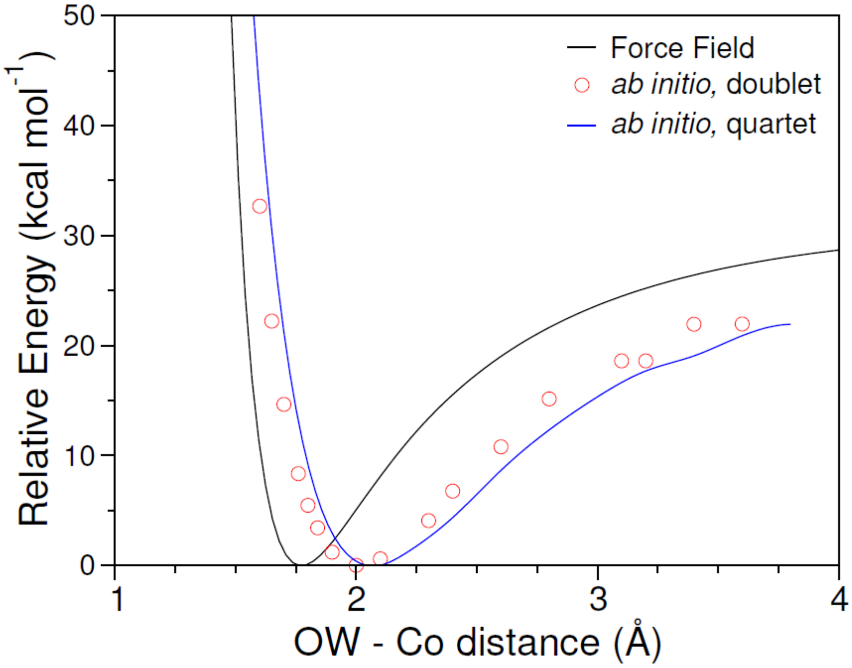

Supplementary Figure 5. Force field development for Co_2Cl_2BTDD Comparison of force field and *ab initio* DFT energies using the *ω*B97X-D functional in combination with the LanL2DZ basis set of both the doublet and quartet spin states on the Co^{2+} atom of the reduced molecular model as in **Supplementary Figure 1**. A water molecule was scanned relative to the Co^{2+} atom of the framework for relative energies up to 50 kcal mol^{-1}.

Theoretical IR spectra of water (framework vibrations neglected) for all loadings were calculated using

$$I_{IR} = \left[\frac{2\omega}{3V\hbar c\epsilon_0}\right] \tanh(\beta\hbar\omega) \int_{-\infty}^{\infty} e^{i\omega t} \langle \mu(0)\mu(t) \rangle \, dt \qquad (1)$$

in the time-dependent formalism where V is the system volume, c is the speed of light in vacuum, ϵ_o is the permittivity of free space, and $\beta = (k_B T)^{-1}$ with k_B being Boltzmann's constant. In eq. 1, $\langle \mu(0)\mu(t) \rangle$ is the ensemble-averaged quantum dipole—dipole time correlation function calculated by averaging $\mu(0)\mu(t)$ over all the MB-MD trajectories at each loading. The dipole moment is given as the many-body dipole moment MB-μ, which contains explicit terms for the one-body (1B) and two-body (2B) terms.[17] All higher-order N-body (NB) terms are represented by classical induction. Classical MB-MD simulations with MB-pol[3-5] have been previously shown to underestimate anharmonic effects due to neglect of zero-point energy.[18] As a result, all theoretical spectra are red-shifted by 60 cm^{-1} and 175 cm^{-1} in the bending and stretching regions, respectively, to account for nuclear quantum effects.[17,19]

The density of states (DOS) spectra were calculated using[20]

$$I_{DOS} = \int_{-\infty}^{\infty} e^{i\omega t} \langle v(0)v(t) \rangle \, dt \qquad (2)$$

in the time-dependent formalism. In eq. 2, $\langle v(0)v(t)\rangle$ is the ensemble-averaged classical velocity-velocity autocorrelation function calculated by averaging $v(0)v(t)$ over all the MD trajectories at each loading. Frequencies from the DOS spectrum can be compared to the IR spectrum, but since the velocity—velocity autocorrelation function is not weighted by the dipole moments, the intensities cannot be compared between the two. The DOS spectra are again shifted by 175 cm^{-1}. The orientational correlation functions were calculated using[21]

$$C_2(t) = \langle P_2[e(0) \cdot e(t)]\rangle \qquad (3)$$

where $P_2[e(0)e(t)]$ is the second Legendre polynomial of the angle formed by the unit vector $e(t)$ that lies along one of the OH bonds of a water molecule. The angle brackets indicate an ensemble average of OH bonds over time. The diffusion coefficients were calculated using[20]

$$D = \frac{1}{3}\int_0^\infty \langle v(0)v(t)\rangle \, dt \qquad (4)$$

where $\langle v(0)v(t)\rangle$ is the ensemble-averaged classical velocity-velocity autocorrelation function. The limit of the autocorrelation function at long times is taken to determine the total diffusion coefficient as well as the diffusion coefficient in the *xy*- and *z*-planes. We extended our 50 ps simulations to 100 ps to ensure convergence of our dynamical properties, and after this extension, we still saw long relaxation times for the confined water. Order parameters describe how ordered a liquid is and are calculated as functions of the spherical harmonics.[22] The orientational order parameter was calculated using[23]

$$q_i = 1 - \frac{3}{8}\sum_{j=1}^{3}\sum_{k=j+1}^{4}\left(\cos\psi_{ijk} + \frac{1}{3}\right)^2 \qquad (5)$$

where ψ_{ijk} is the angle formed by the oxygen atom on water molecule *i* and the oxygen atoms of two neighboring water molecules *j* and *k* at a distance less than 3.5 Å. Specifically, the tetrahedral order parameter utilized here relates the probability of a liquid being tetrahedral by looking at a molecule's four nearest neighbors based on distance. An order parameter close to zero suggests that the liquid is not tetrahedral as in an ideal gas, and an order parameter close to one suggests that the liquid is perfectly tetrahedral.[23]

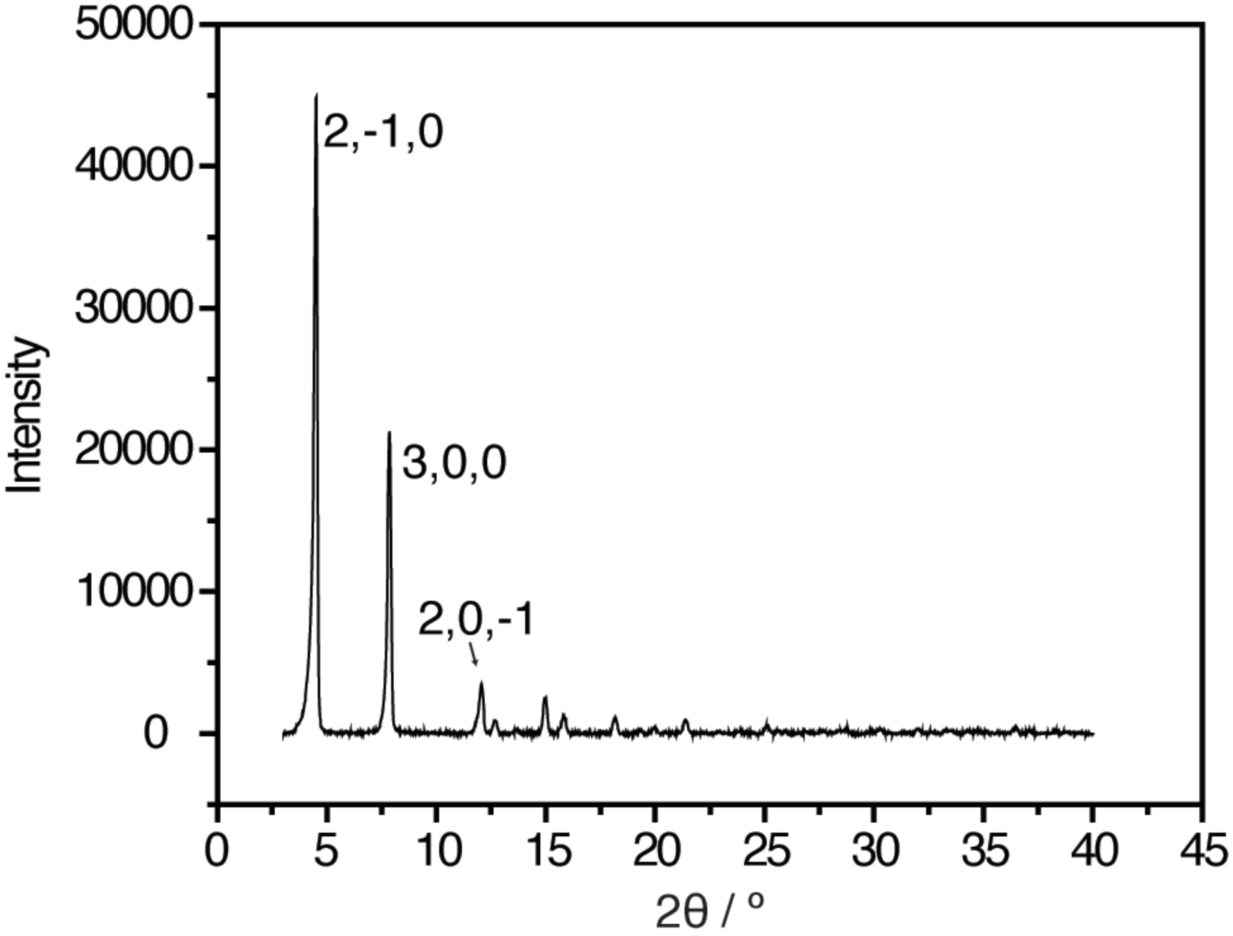

Supplementary Figure 6. X-ray diffraction Powder X-ray diffraction pattern of as-synthesized Co_2Cl_2BTDD, with Miller indices of the initial 3 reflections labeled.

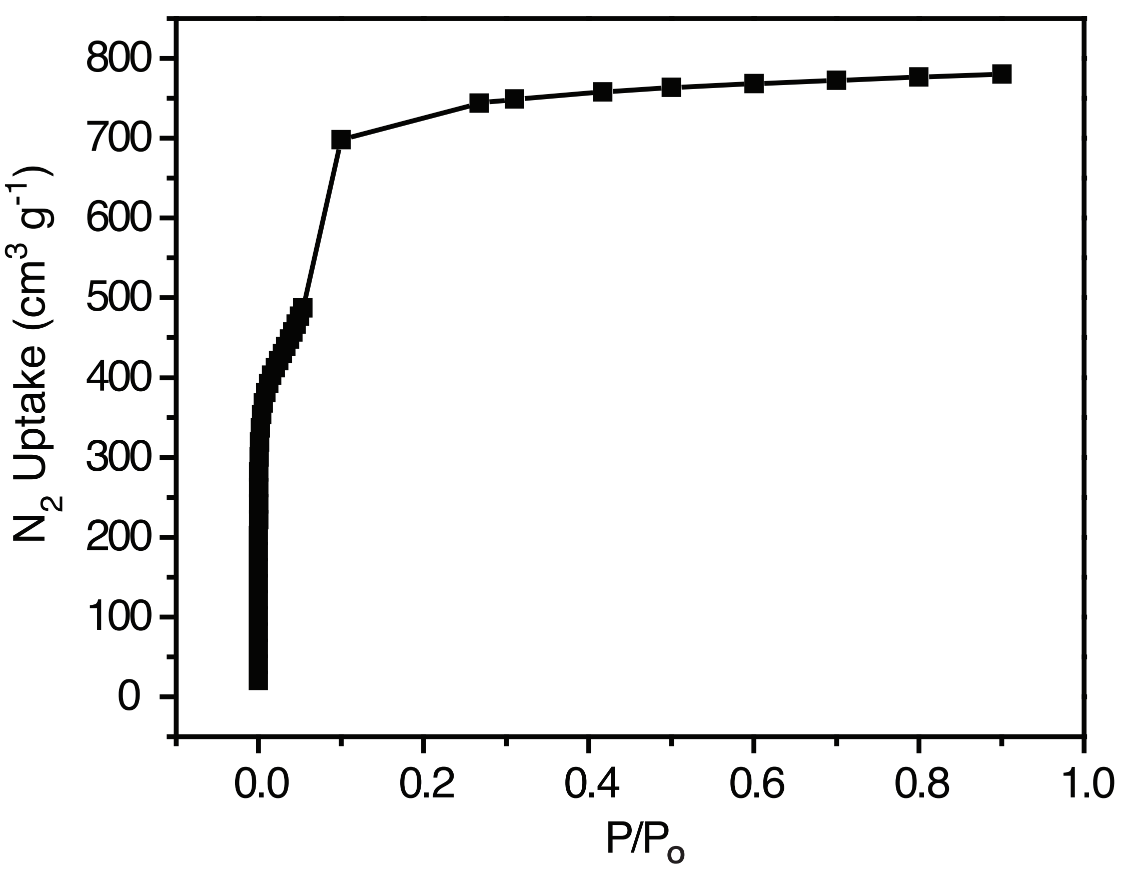

Supplementary Figure 7. Adsorption Isotherm N_2 adsorption isotherm at 77 K of an activated sample of Co_2Cl_2BTDD. Calculated BET surface area 1905 m^2 g^{-1}.

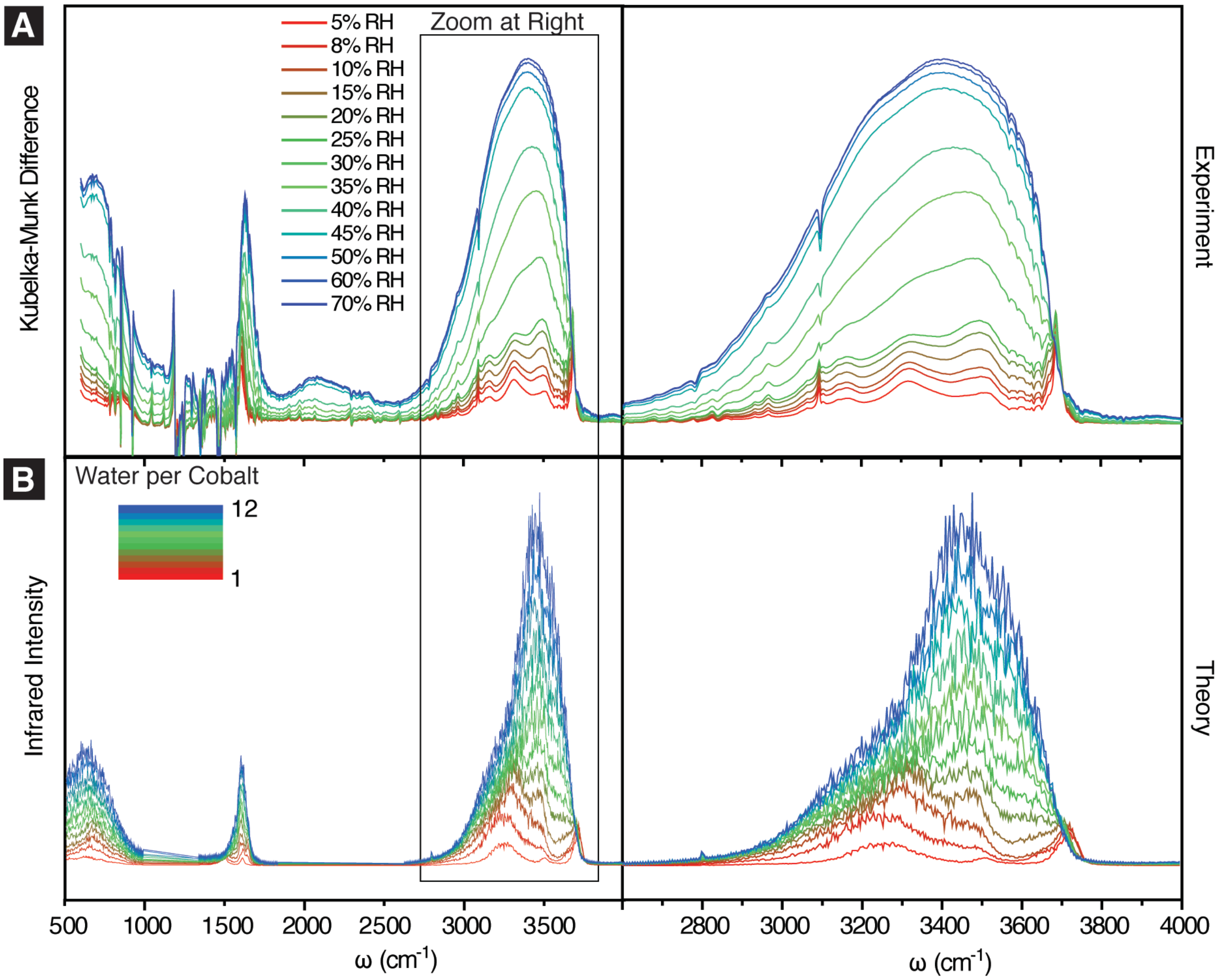

Supplementary Figure 8. Experimental and Theoretical Infrared Spectra of Water in Co_2Cl_2BTDD A) Difference Diffuse-Reflectance IR spectrum (left) and zoom in on the water OH stretch region (right) in Co_2Cl_2BTDD under variable humidity conditions. Because these are difference spectra, anomalous features appearing as sharp spikes or negative peaks correspond to frequencies of dry framework IR absorption bands. B) Calculated infrared intensity using the theoretical MB-pol model of water in Co_2Cl_2BTDD corresponding to one water molecule per cobalt (1) up to twelve water molecules per cobalt (12).

Supplementary Table 5: Comparison of Experimental and Theoretical Frequencies Frequencies of maximum absorbance for the four main peaks plus the shoulder of the highest-frequency peak (~3670 cm^{-1}) for the experimental DRIFTS spectra at 20% RH (**Figure 2A** and **Supplementary Figure 5**) and the theoretical density of states (**Figure 4B**) at a loading of two water molecules per cobalt. All frequencies are given in wavenumbers (cm^{-1}).

Experiment 20% RH	Theoretical density of states, 2 H_2O / Co^{2+}
3161	3065
3323	3332
3492	3485
3667	3679
3684	3709

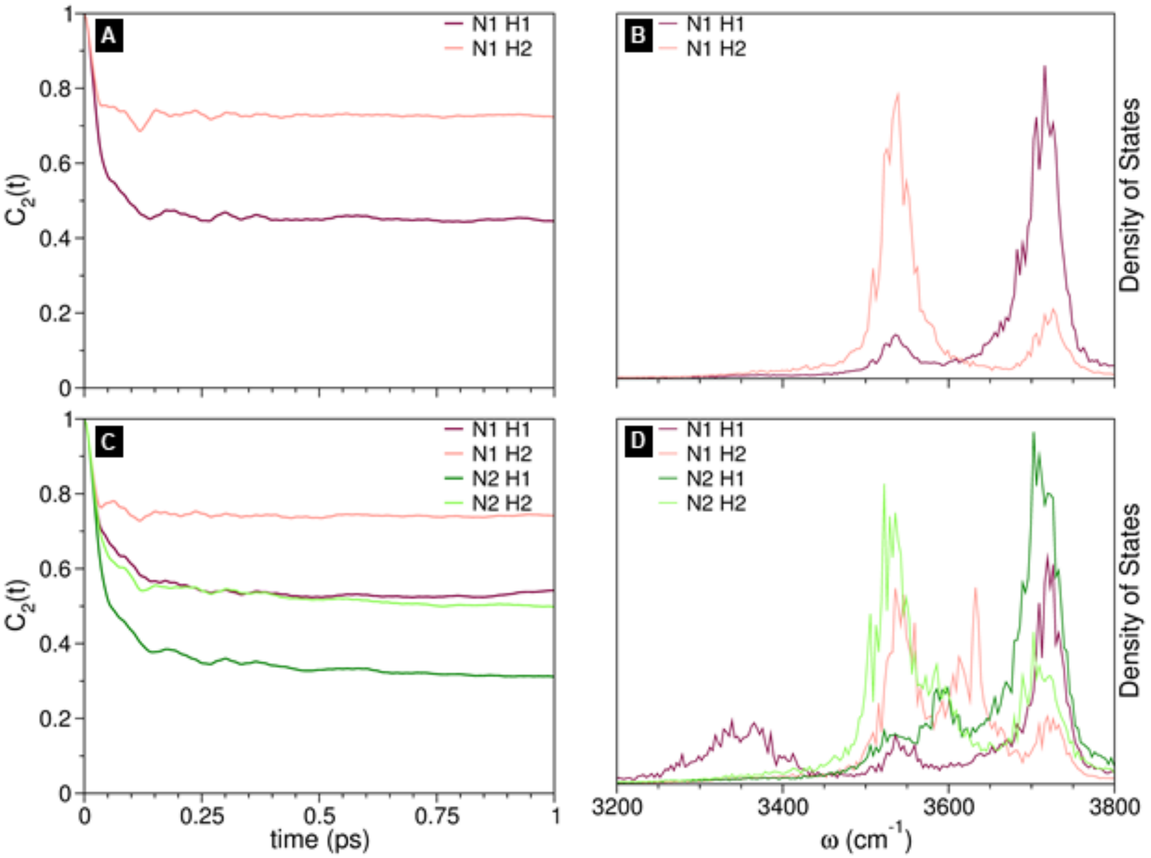

Supplementary Figure 9. Structure and Dynamics of Water in Co_2Cl_2BTDD Orientational correlation functions and density of states of individual hydrogen atoms in the one- and two-water molecule simulations. "N" denotes which water molecule is under investigation, and "H" denotes which hydrogen atom of the current water molecule is used in the calculation. A) Correlation function for the single water molecule simulation. B) Density of states for the single water molecule simulation. C) Correlation function for the two-water molecule simulation. D) Density of states for the two-water molecule simulation.

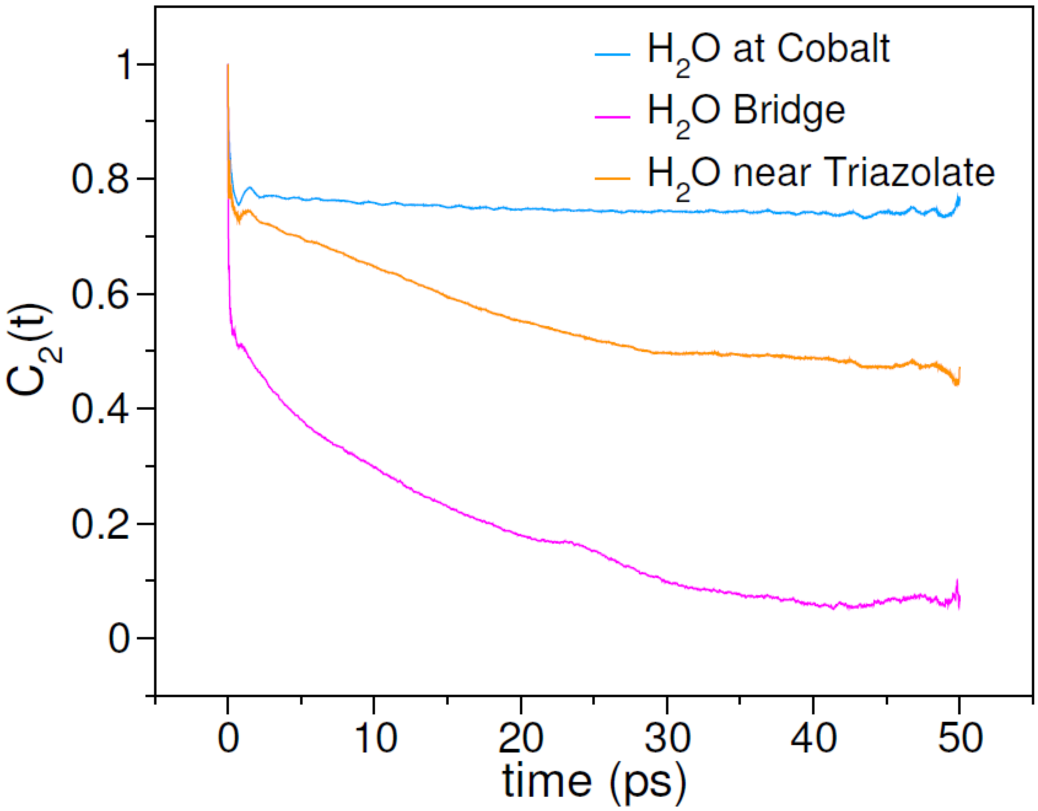

Supplementary Figure 10. Correlation functions Orientational correlation function for water molecules in different H-bond environments of the 1-D chain.

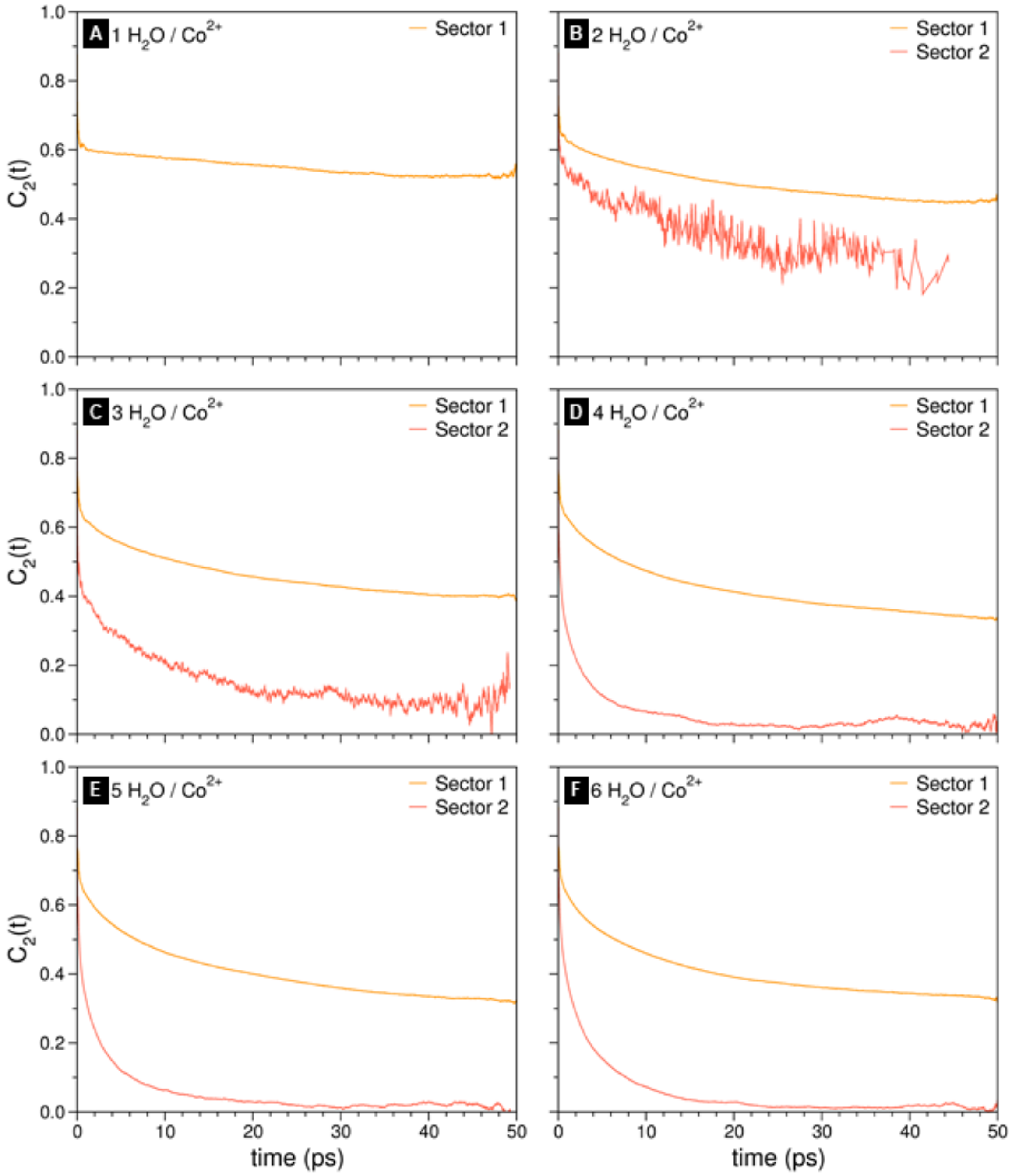

Supplementary Figure 11. Dynamics in Different Regions Orientational correlation functions of water inside the three different sectors of the pore at all loadings. Sector 1 is 0 – 4 Å from the surface of the pore (dark yellow), sector 2 is 4 – 8 Å from the surface of the pore (orange), and sector 3 is 8 –12 Å from the surface of the pore (red).

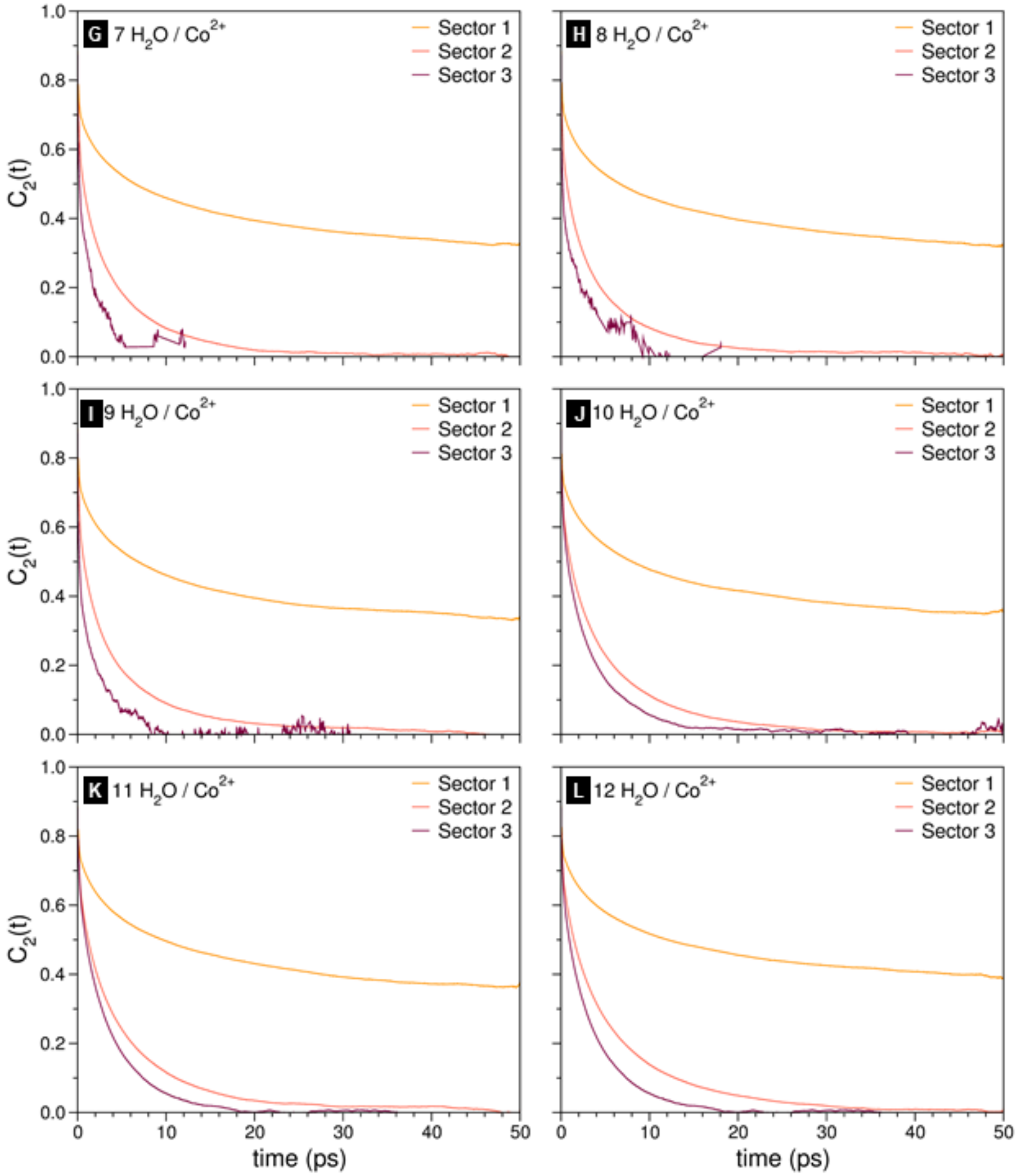

Supplementary Figure 12. Dynamics in Different Regions Orientational correlation functions of water inside the three different sectors of the pore at all loadings. Sector 1 is 0 – 4 Å from the surface of the pore (dark yellow), sector 2 is 4 – 8 Å from the surface of the pore (orange), and sector 3 is 8 –12 Å from the surface of the pore (red).

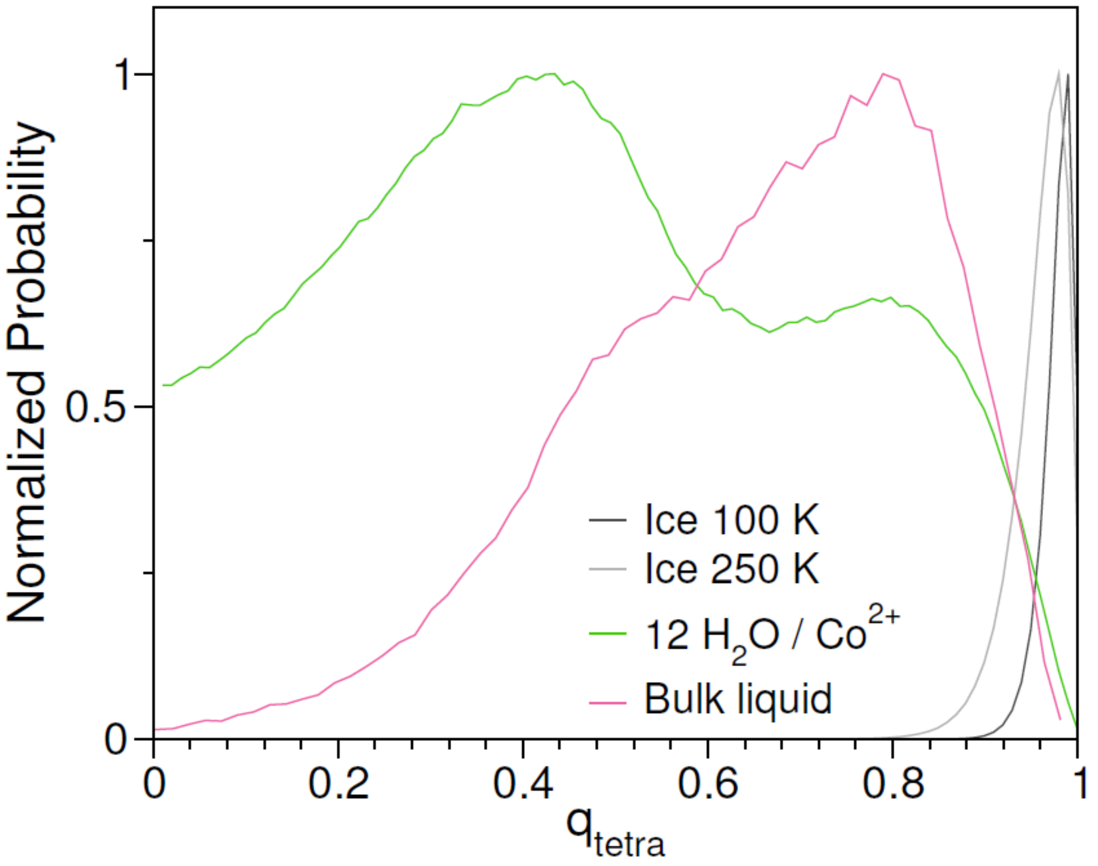

Supplementary Figure 13. Confined Water Structure Compared to Ice and Bulk Liquid Water Tetrahedral order parameters for the highest loading. An order parameter closer to zero indicates a gas-like molecule with a less tetrahedral structure, and an order parameter closer to one indicates an ice-like molecule with a more tetrahedral arrangement.

Supplementary Table 6: Simulation Temperature Average temperature and associated standard deviation for each of the 20 trajectories for a loading of 1 H_2O / Co^{2+}. Also listed is the average temperature and associated standard deviation calculated over all 20 trajectories.

Trajectory	Average temperature (K)
1	304 ± 6
2	310 ± 6
3	303 ± 6
4	305 ± 6
5	303 ± 6
6	294 ± 6
7	298 ± 6
8	293 ± 6
9	305 ± 6
10	294 ± 6
11	304 ± 6
12	299 ± 6
13	299 ± 6
14	302 ± 6
15	299 ± 6
16	297 ± 6
17	288 ± 6
18	299 ± 6
19	299 ± 6
20	301 ± 6
average	300 ± 6

Supplementary Table 7: Simulation Temperature Average temperature and associated standard deviation for each of the 20 trajectories for a loading of 2 H_2O / Co^{2+}. Also listed is the average temperature and associated standard deviation calculated over all 20 trajectories.

Trajectory	Average temperature (K)
1	300 ± 6
2	297 ± 5
3	300 ± 5
4	297 ± 5
5	304 ± 5
6	312 ± 6
7	304 ± 6
8	299 ± 5
9	302 ± 5
10	301 ± 5
11	293 ± 5
12	302 ± 5
13	306 ± 5
14	300 ± 5
15	301 ± 5
16	303 ± 5
17	301 ± 5
18	300 ± 5
19	296 ± 5
20	297 ± 5
average	301 ± 5

Supplementary Table 8: Simulation Temperature Average temperature and associated standard deviation for each of the 20 trajectories for a loading of 3 H_2O / Co^{2+}. Also listed is the average temperature and associated standard deviation calculated over all 20 trajectories.

Trajectory	Average temperature (K)
1	311 ± 6
2	299 ± 5
3	302 ± 5
4	299 ± 5
5	300 ± 5
6	294 ± 5
7	300 ± 5
8	299 ± 5
9	301 ± 5
10	296 ± 5
11	303 ± 5
12	305 ± 5
13	298 ± 5
14	302 ± 5
15	298 ± 5
16	303 ± 5
17	303 ± 5
18	307 ± 5
19	299 ± 5
20	305 ± 5
average	301 ± 5

Supplementary Table 9: Simulation Temperature Average temperature and associated standard deviation for each of the 20 trajectories for a loading of 4 H_2O / Co^{2+}. Also listed is the average temperature and associated standard deviation calculated over all 20 trajectories.

Trajectory	Average temperature (K)
1	318 ± 6
2	300 ± 5
3	294 ± 5
4	305 ± 5
5	302 ± 5
6	300 ± 5
7	295 ± 5
8	297 ± 5
9	297 ± 5
10	312 ± 5
11	291 ± 5
12	302 ± 5
13	301 ± 5
14	303 ± 5
15	302 ± 5
16	301 ± 5
17	295 ± 5
18	298 ± 5
19	303 ± 5
20	295 ± 5
average	301 ± 5

Supplementary Table 10: Simulation Temperature Average temperature and associated standard deviation for each of the 20 trajectories for a loading of 5 H_2O / Co^{2+}. Also listed is the average temperature and associated standard deviation calculated over all 20 trajectories.

Trajectory	Average temperature (K)
1	308 ± 5
2	304 ± 5
3	296 ± 5
4	301 ± 5
5	299 ± 5
6	299 ± 5
7	304 ± 5
8	295 ± 5
9	300 ± 5
10	304 ± 5
11	303 ± 5
12	306 ± 5
13	306 ± 5
14	303 ± 5
15	293 ± 5
16	304 ± 5
17	303 ± 5
18	297 ± 5
19	298 ± 5
20	297 ± 5
average	301 ± 5

Supplementary Table 11: Simulation Temperature Average temperature and associated standard deviation for each of the 20 trajectories for a loading of 6 H_2O / Co^{2+}. Also listed is the average temperature and associated standard deviation calculated over all 20 trajectories.

Trajectory	Average temperature (K)
1	310 ± 5
2	303 ± 5
3	299 ± 5
4	300 ± 5
5	297 ± 5
6	304 ± 5
7	302 ± 5
8	304 ± 5
9	297 ± 5
10	305 ± 5
11	301 ± 5
12	298 ± 5
13	300 ± 4
14	298 ± 4
15	299 ± 5
16	298 ± 5
17	304 ± 5
18	299 ± 5
19	293 ± 4
20	294 ± 4
average	300 ± 5

Supplementary Table 12: Simulation Temperature Average temperature and associated standard deviation for each of the 20 trajectories for a loading of 7 H_2O / Co^{2+}. Also listed is the average temperature and associated standard deviation calculated over all 20 trajectories.

Trajectory	Average temperature (K)
1	306 ± 5
2	304 ± 4
3	306 ± 5
4	299 ± 4
5	304 ± 4
6	300 ± 4
7	298 ± 4
8	300 ± 4
9	302 ± 4
10	302 ± 4
11	306 ± 5
12	297 ± 4
13	300 ± 5
14	296 ± 4
15	306 ± 5
16	297 ± 4
17	301 ± 4
18	299 ± 5
19	304 ± 4
20	298 ± 4
average	301 ± 4

Supplementary Table 13: Simulation Temperature Average temperature and associated standard deviation for each of the 20 trajectories for a loading of 8 H_2O / Co^{2+}. Also listed is the average temperature and associated standard deviation calculated over all 20 trajectories.

Trajectory	Average temperature (K)
1	305 ± 5
2	302 ± 4
3	299 ± 4
4	299 ± 4
5	296 ± 4
6	301 ± 4
7	295 ± 4
8	305 ± 4
9	298 ± 4
10	301 ± 4
11	302 ± 4
12	303 ± 4
13	300 ± 4
14	298 ± 4
15	298 ± 4
16	302 ± 4
17	298 ± 4
18	300 ± 4
19	298 ± 4
20	297 ± 4
average	300 ± 4

Supplementary Table 14: Simulation Temperature Average temperature and associated standard deviation for each of the 20 trajectories for a loading of 9 H_2O / Co^{2+}. Also listed is the average temperature and associated standard deviation calculated over all 20 trajectories.

Trajectory	Average temperature (K)
1	302 ± 4
2	303 ± 4
3	300 ± 4
4	300 ± 4
5	296 ± 4
6	299 ± 4
7	298 ± 4
8	296 ± 4
9	298 ± 4
10	298 ± 4
11	301 ± 4
12	298 ± 4
13	307 ± 4
14	303 ± 4
15	299 ± 4
16	300 ± 4
17	299 ± 4
18	300 ± 4
19	303 ± 4
20	301 ± 4
average	300 ± 4

Supplementary Table 15: Simulation Temperature Average temperature and associated standard deviation for each of the 20 trajectories for a loading of 10 H_2O / Co^{2+}. Also listed is the average temperature and associated standard deviation calculated over all 20 trajectories.

Trajectory	Average temperature (K)
1	308 ± 4
2	299 ± 4
3	305 ± 4
4	306 ± 4
5	299 ± 4
6	300 ± 4
7	297 ± 4
8	300 ± 4
9	301 ± 4
10	300 ± 4
11	298 ± 4
12	300 ± 4
13	298 ± 4
14	303 ± 4
15	299 ± 4
16	299 ± 4
17	301 ± 4
18	296 ± 4
19	302 ± 4
20	301 ± 4
average	301 ± 4

Supplementary Table 16: Simulation Temperature Average temperature and associated standard deviation for each of the 20 trajectories for a loading of 11 H_2O / Co^{2+}. Also listed is the average temperature and associated standard deviation calculated over all 20 trajectories.

Trajectory	Average temperature (K)
1	306 ± 4
2	310 ± 4
3	305 ± 4
4	305 ± 4
5	299 ± 4
6	298 ± 4
7	304 ± 4
8	298 ± 4
9	301 ± 4
10	298 ± 4
11	301 ± 4
12	302 ± 4
13	298 ± 4
14	301 ± 4
15	301 ± 4
16	300 ± 4
17	302 ± 4
18	301 ± 4
19	300 ± 4
20	303 ± 4
average	302 ± 4

Supplementary Table 17: Simulation Temperature Average temperature and associated standard deviation (in K) for each of the 20 trajectories for a loading of 12 H_2O / Co^{2+}. Also listed is the average temperature and associated standard deviation calculated over all 20 trajectories.

Trajectory	Average temperature (K)
1	302 ± 4
2	301 ± 4
3	298 ± 4
4	301 ± 4
5	298 ± 4
6	299 ± 4
7	299 ± 4
8	300 ± 4
9	299 ± 4
10	302 ± 4
11	300 ± 4
12	297 ± 4
13	297 ± 4
14	301 ± 4
15	302 ± 4
16	299 ± 4
17	293 ± 4
18	301 ± 4
19	301 ± 4
20	298 ± 4
average	299 ± 4

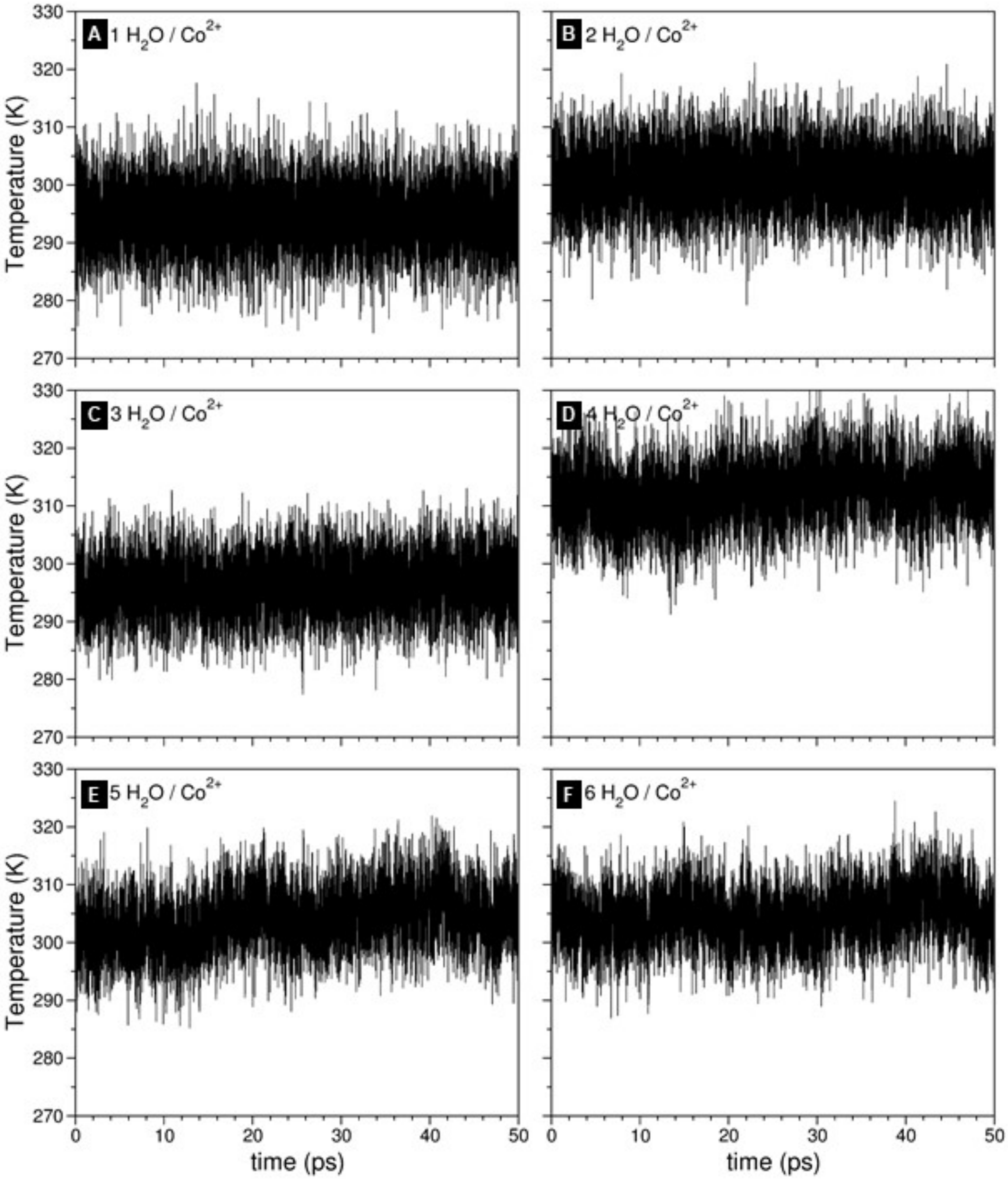

Supplementary Figure 14. Simulation Temperature Temperature of the 10th NVE trajectory for loadings of 1 to 6 H_2O / Co^{2+}. The average temperature and associated standard deviation of all 20 NVE trajectories for loadings of 1 to 6 H_2O / Co^{2+} are listed in Supplementary Tables 6-11.

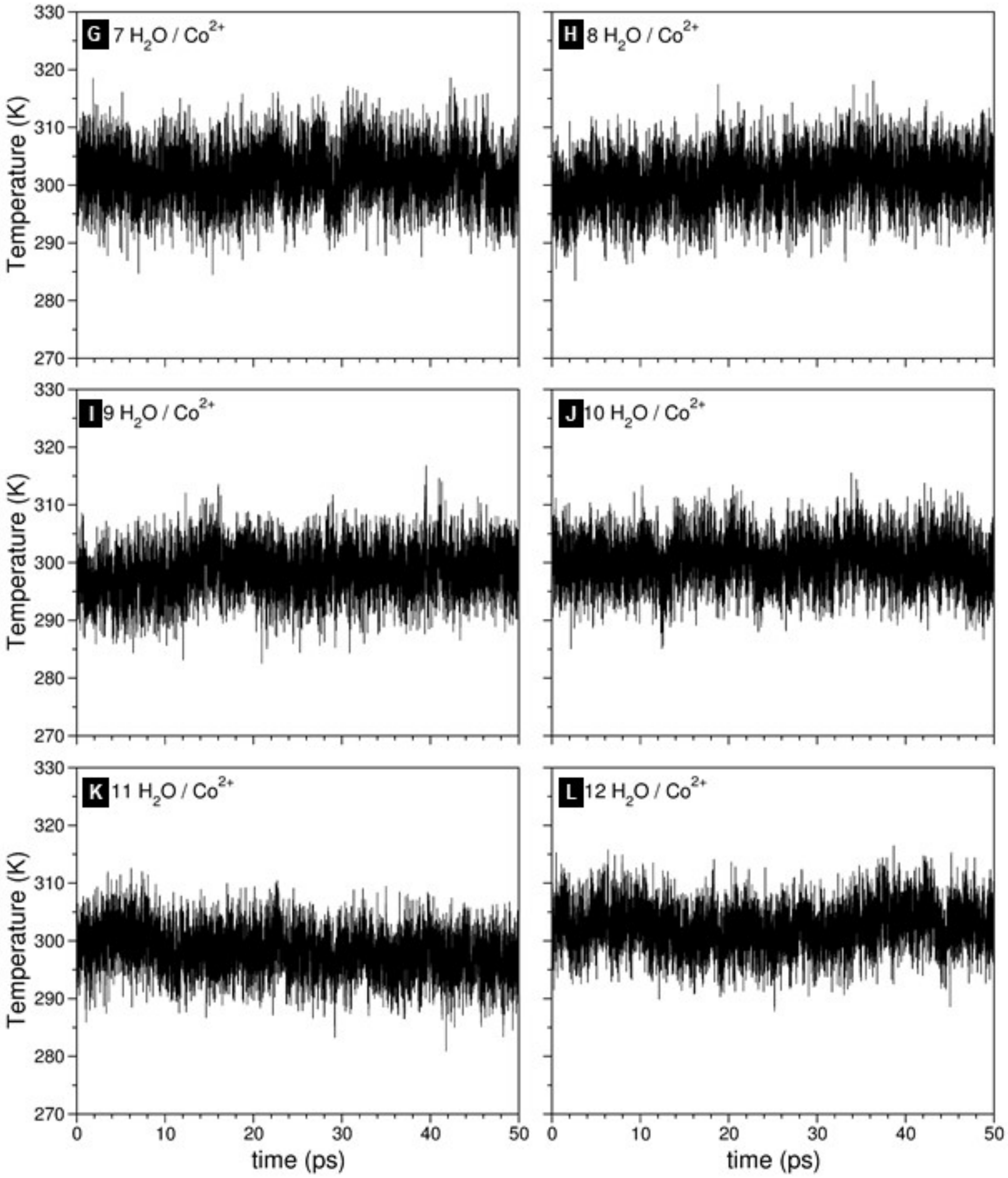

Supplementary Figure 15. Simulation Temperature Temperature of the 10th NVE trajectory for loadings of 7 to 12 H_2O / Co^{2+}. The average temperature and associated standard deviation of all 20 NVE trajectories for loadings of 7 to 12 H_2O / Co^{2+} are listed in Supplementary Tables 12-17.

Supplementary References:

1 Martinez, L., Andrade, R., Birgin, E. G. & Martinez, J. M. PACKMOL: A Package for Building Initial Configurations for Molecular Dynamics Simulations. *J. Comput. Chem.* **30**, 2157 (2009).

2 Smith, W. & Forester, T. R. DL_POLY_2.0: A general-purpose parallel molecular dynamics simulation package. *J. Mol. Graph.* **14**, 136-141 (1996).

3 Babin, V., Leforestier, C. & Paesani, F. Development of a "first principles" water potential with flexible monomers: dimer potential energy surface, VRT spectrum, and second virial coefficient. *J. Chem. Theory. Comput.* **9**, 5395 (2013).

4 Babin, V., Medders, G. R. & Paesani, F. Development of a "first principles" water potential with flexible monomers. II: Trimer potential energy surface, third virial coefficient, and small clusters. *J. Chem. Theory. Comput.* **10**, 1599-1607 (2014).

5 Medders, G. R., Babin, V. & Paesani, F. Development of a "first-principles" water potential with flexible monomers. III. Liquid phase properties. *J. Chem. Theory. Comput.* **10**, 2906-2910 (2014).

6 Tuckerman, M. E. *Statistical Mechanics: Theory and Molecular Simulation*. (Oxford University Press, 2010).

7 Leach, A. R. *Molecular Modeling: Principles and Applications*. (Pearson Prentice Hall, 2001).

8 Wang, J. M., Wolf, R. M., Caldwell, J. W., Kollman, P. A. & Case, D. A. Development and testing of a general amber force field. *J. Comput. Chem.* **25**, 1157-1174 (2004).

9 Gaussian 09 (Gaussian, Inc., Wallingford, CT, USA, 2009).

10 Chai, J. D. & Head-Gordon, M. Long-range corrected hybrid density functionals with damped atom-atom dispersion corrections. *Phys. Chem. Chem. Phys.* **10**, 6615-6620 (2008).

11 Dunning, T. H. & Hay, P. J. in *Modern Theoretical Chemistry* Vol. 3 (ed H. F. Schaefer) 1-28 (Plenum, 1977).

12 Hay, P. J. & Wadt, W. R. Ab initio effective core potentials for molecular calculations - Potentials for the transition-metal atoms Sc to Hg. *J. Chem. Phys.* **82**, 270 (1985).

13 Wadt, W. R. & Hay, P. J. Ab initio effective core potentials for molecular calculations - Potentials for main group elements Na to Bi. *J. Chem. Phys.* **82**, 284 (1985).

14 Hay, P. J. & Wadt, W. R. Ab initio effective core potentials for molecular calculations - Potentials for K to Au including the outermost core orbitals. *J. Chem. Phys.* **82**, 299 (1985).

15 Goldberg, D. E. *Genetic Algorithms in Search, Optimization and Machine Learning*. (Addison-Wesley Longman Publishing Co., Inc., 1989).

16 Abascal, J. L. F. & Vega, C. A general purpose model for the condensed phases of water: TIP4P/2005. *J. Chem. Phys.* **123**, 234505 (2005).

17 Medders, G. R. & Paesani, F. Infrared and Raman spectroscopy of liquid water through "first-principles" many-body molecular dynamics. *J. Chem. Theory. Comput.* **11**, 1145 (2015).

18 Reddy, S. K., Moberg, D. R., Straight, S. C. & Paesani, F. Temperature-dependent vibrational spectra and structure of liquid water from classical and quantum simulations with the MB-pol potential energy function. *J. Chem. Phys.* **147**, 244504 (2017).

19 Medders, G. R. & Paesani, F. Dissecting the molecular structure of the air/water interface from quantum simulations of the sum-frequency generation spectrum. *J. Am. Chem. Soc.* **138**, 3912 (2016).

20 Nitzan, A. *Chemical dynamics in condensed phases: relaxation, transfer and reactions in condensed molecular systems*. (Oxford University Press, 2006).

21 Bakker, H. J. & Skinner, J. L. Vibrational spectroscopy as a probe of structure and dynamics in liquid water. *Chem. Rev.* **110**, 1498 (2010).

22 Steinhardt, P. J., Nelson, D. R. & Ronchetti, M. Bond-orientational order in liquids and glasses. *Phys. Rev. B* **28**, 784 (1983).

23 Errington, J. R. & Debenedetti, P. G. Relationship between structural order and the anomalies of liquid water. *Nature* **409**, 318 (2001).
